# Experimental single-strain mobilomics reveals events that shape pathogen emergence

**DOI:** 10.1093/nar/gkw601

**Published:** 2016-07-04

**Authors:** Joseph S. Schoeniger, Corey M. Hudson, Zachary W. Bent, Anupama Sinha, Kelly P. Williams

**Affiliations:** Systems Biology Department, Sandia National Laboratories, Livermore, CA 94551, USA

## Abstract

Virulence genes on mobile DNAs such as genomic islands (GIs) and plasmids promote bacterial pathogen emergence. Excision is an early step in GI mobilization, producing a circular GI and a deletion site in the chromosome; circular forms are also known for some bacterial insertion sequences (ISs). The recombinant sequence at the junctions of such circles and deletions can be detected sensitively in high-throughput sequencing data, using new computational methods that enable empirical discovery of mobile DNAs. For the rich mobilome of a hospital *Klebsiella pneumoniae* strain, circularization junctions (CJs) were detected for six GIs and seven IS types. Our methods revealed differential biology of multiple mobile DNAs, imprecision of integrases and transposases, and differential activity among identical IS copies for IS*26*, IS*Kpn18* and IS*Kpn21*. Using the resistance of circular dsDNA molecules to exonuclease, internally calibrated with the native plasmids, showed that not all molecules bearing GI CJs were circular. Transpositions were also detected, revealing replicon preference (IS*Kpn18* prefers a conjugative IncA/C_2_ plasmid), local action (IS*26*), regional preferences, selection (against capsule synthesis) and IS polarity inversion. Efficient discovery and global characterization of numerous mobile elements per experiment improves accounting for the new gene combinations that arise in emerging pathogens.

## INTRODUCTION

Bacterial pathogens acquire many of their antibiotic resistance and virulence genes as cargo on mobile elements that transfer between cells, typically through conjugation tubes or phage particles. Once delivered to recipient cells, these DNAs circularize and are then stably maintained either by self-replicating as plasmids or by integrating into the chromosome as genomic islands (GIs). Other mobile elements that move only intracellularly, such as transposons, are nonetheless relevant to pathogen emergence because they can insert into, and thereby augment the cargos of, the inter-bacterial mobile DNAs.

**Figure 1.**
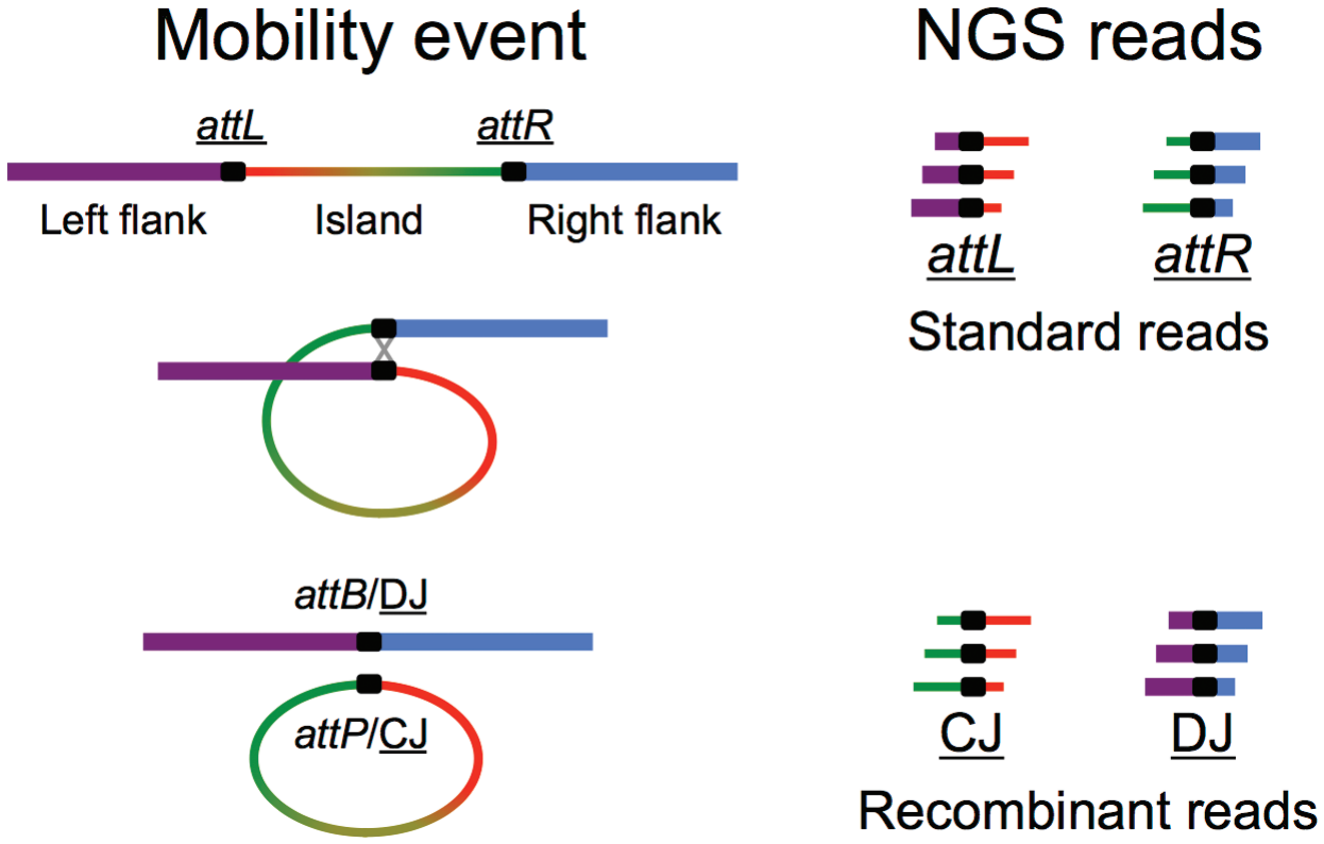
Detecting excision products by NGS. Island excision is shown, but some ISs circularize also, hence the general terms CJ (circularization junction) and DJ (deletion junction) for excision products. Island excision produces stoichiometric amounts of CJ and DJ, but IS circularization produces a CJ only. The black segment represents the sequence-identical overlap region in which crossover takes place.

While plasmids are readily identified in genome projects by their circular assembly maps, GIs can be more challenging to delineate. A classical GI is the prophage form of bacteriophage lambda. Phage-encoded integrase catalyzes crossover between the phage *attP* site and the related *attB* chromosomal target site, leaving the prophage flanked by the recombinant *attL* and *attR* sites (1). Upon induction of the lysogen, the reverse excision reaction leaves (i) circular lambda DNA whose circularization junction (CJ) is the regenerated *attP* and (ii) a deletion junction (DJ) in the chromosome, the regenerated *attB* (Figure 1). Some bacterial ISs are likewise known to form intermediates with CJs (2–4). Excised phage lambda goes on to replicate intracellularly (5).

Several complementary bioinformatic approaches to GI discovery take advantage of common but non-universal island features: (i) their preference for target sites in tRNA and tmRNA genes (6), (ii) their density of phage-associated genes (7), (iii) anomalous nucleotide composition (8) and (iv) sporadic occurrence among closely related strains (9). None of these approaches can find all islands, precisely define their genomic coordinates or guarantee that those found have retained their mobility. Experimental approaches to discovery of active islands often employ the DNA-damaging agent mitomycin C (MMC) as an inducer. The released phages may then be isolated and characterized, but this approach misses non-viral islands like integrative conjugative elements. Predicted elements can be validated experimentally by inducing and detecting with polymerase chain reaction (PCR) the *attP* of the excised island; however, for some of the bioinformatic approaches the predictions are sufficiently inaccurate that PCR test designs may fail.

We present a new experimental approach for the discovery of multiple islands, tranposable elements and (in principle) other mobile DNAs, using high-throughput (next-generation) sequencing (NGS) to map them onto the genome with nucleotide precision. This approach is an unbiased discovery method, that relies on no preconceptions about the nature of the mobile DNA. The data from our method inform questions about the biased distributions of transposition events, mechanisms of DNA mobility and comparative biology of multiple mobile DNAs.

## MATERIALS AND METHODS

### Culture

A stock of *Klebsiella pneumoniae* BAA-2146 (Kpn2146) was obtained from American Type Culture Collection (ATCC). Its minimum inhibitory concentration of MMC was measured by broth microdilution at 6 μg/ml. For each experiment, a Kpn2146 colony on an LB agar plate was inoculated into LB broth and shaken at 37°C overnight. Shaking was continued for this overnight culture, and it was also diluted 100-fold into fresh broth and grown to log phase (OD600 = 0.5). At this point, MMC was added to cultures at a low (1 μg/ml) or high (5 μg/ml) level and incubation was continued for indicated periods before harvesting cells.

### Sequencing

‘Experiment X0’ sequencing data from Kpn2146 genomic DNA purchased from ATCC was described previously (10); read pairs were merged as possible using PEAR, and merged and unmergeable sequences were pooled. For the new experiments (Table 3), genomic DNA was extracted and purified from culture using DNeasy blood and tissue kit (Qiagen) as instructed. Nanodrop 2000 and Qubit DNA high sensitivity quantification kit was used to check quality and quantity. Aliquots (1 μg DNA) of some samples were treated with the exonucleolytic Plasmid-Safe DNase (Epicentre), in 50 μL volumes of the supplied buffer with 10 units DNase, incubated overnight at 37°C. Sequencing libraries were prepared using Illumina Nextera DNA sample preparation kit as instructed. Libraries were sequenced (single end, 150 cycle) on Illumina NextSeq 500 in high output mode or MiSeq. A quality filter (10) was applied to raw read data.

### Bioinformatics

The Juxtaposer software used to find recombination junctions in NGS data is described in SI (‘Juxtaposer software’). Its main download site is bioinformatics.sandia.gov, with source code also available at github.com/sandialabs/Juxtaposer. A first iteration of its use with Kpn2146 identified errors in the genome sequence that were corrected (SI, ‘Genome reassembly’). Once mobile element termini were determined by Juxtaposer, abundance of each element was measured in each sample as the average count for its genome-unique 21-mers among the reads. Additionally, regular expressions (regexes) were designed to distinguish and count *attL, attR, attB* and *attP* for each element, with wild card positions that allow any sequence to intervene between IS ends in CJs. AttCt software applies these regexes and their reverse complements to each read, reporting the matching sequences and summary read counts. The above measurements, for each mobile DNA in each sample, were normalized using **F**, the average counts for the unique 21-mers in 30 kbp at each flank. This normalization factor was more reliable than the average of *attL+attB* and *attR+attB* counts; mean max/min was 1.41 for the latter two counts (when both ≥10), while that for **F** was 1.17, 1.13 and 1.09 using 3, 15 and 30 kbp flanks respectively. attCt/**F** values, for CJs or for DJs, would have a maximum of one if only excision occurs, however replication of CJ forms could take this value higher. Treatment of exonuclease data is described in SI (‘Exonuclease treatment’).

## RESULTS

### NGS monitors DNA mobility that occurs during culture

As part of our original genome project for Kpn2146 we had bioinformatically predicted numerous mobile elements (Tables 1 and 2) in addition to its four plasmids (10). Moreover, while characterizing the newly discovered insertion sequence IS*Kpn21*, we had found that some Illumina reads were from the junction region of circularized forms of the IS, indicating that NGS data report on DNA mobility events occurring during culture (Figure 1). Based on this observation new experiments (Table 3) were designed that use MMC to induce excision of GIs, compare log phase to overnight cultures, and use exonuclease treatment to measure dsDNA circular forms. The new experiments are presented along with further analysis of the original sequencing dataset. Details of the results are presented below, but first the NGS methods that demonstrate activity of mobile elements are described.

**Table 1.** Genomic islands of Kpn2146

			Juxtaposer			AttCt		
Island	Length	Type	CJ	DJ	Tp	CJ	DJ	Remap*
Kpn40guaA	40 467	Podo. prophage	845	137	0	1856	222	59,79
Kpn16fis	15 995	Sipho. prophage	21	0	1	344	2	−31,−31
Kpn49R	49 134	Sipho. prophage	25	5	0	232	115	
Kpn37X	36 567	Myo. prophage	200	3	0	649	6	
Kpn38rybB	37 500	Myo. prophage	1	0	0	1	0	
Kpn42yraA	42 389	Myo. prophage	311	212	0	2376	324	3235,6
Kpn11L	11 188	Satell. prophage	0	0	0	0	1	
Kpn21L	21 481	Satell. prophage	1	0	0	1	0	
Kpn23sapBC^†^	23 301	Satell. prophage	217	841	2	414	1680	
Kpn29S	28 544	MobQP ICE	2	2	0	2	4	
Kpn55F	54 900	MPF-G ICE	0	0	0	0	2	

*Remap: rightward shift of the newly determined terminal coordinates (left, right), relative to original island call.

^†^GI Kpn23sapBC was apparently excised in the founding colony of experiment X3, exaggerating the DJ read count sum.

Read counts for mobilizations (CJ, circularization junction; DJ, deletion junction, Tp, transposition) are from two methods, summed over all experiments.

**Table 2.** Transposable elements of Kpn2146

				Juxtaposer			AttCt		
Type	Length	Family	Intact copies	CJ	DJ	Tp	CJ	DJ	Ref*
IS*1F*	768	IS*1*	1	0	0	2	0	0	
IS*1R*	768	IS*1*	1	0	0	0	0	0	
IS*1×4*	768	IS*1*	1	0	0	6	0	0	
IS*Kpn14*	768	IS*1*	2	5	0	115	8	0	
IS*26*	820	IS*6*	11	2	0	170	25	0	(11)
IS*6100*	880	IS*6*	2	0	1	3	0	0	
IS*903B*	1057	IS*5*	2	0	0	0	0	0	
IS*4321*	1327	IS*110*	1	5248	0	5	5241	0	(10)
IS*5075*	1327	IS*110*	1	403	0	0	401	0	(10)
IS*Kpn18*	1303	IS*3*	1	30	1	17	50	0	
IS*Ecl1*	1336	IS*3*	2	0	0	1	0	0	
IS*Kpn1*	1445	IS*3*	1	20	0	1	31	0	
IS*Ecp1*	1656	IS*1380*	1	0	0	3	0	0	
IS*Kpn21*	2278	IS*NCY*	1	194	0	0	447	0	(8)
IS*Ec22*	2454	IS*66*	1	0	0	1	0	0	
IS*3000*	3236	Tn*3*	1	0	0	0	0	0	
Tn*6187*	9308	Tn*3*	1	0	0	1	0	0	

*References to circularization of insertion sequence.

Layout as in Table 1.

**Table 3.** Experiments and samples, with the indicated harvest time after MMC treatment

Experiment	Sample	Growth phase	MMC (μg/ml)	Harvest time (min)
X0	S1	by ATCC*	0	0
X1	S1	log phase	0	15
X1	S2	log phase	1	15
X2	S1	log phase	0	0
X2	S2	log phase	1	60
X2	S3	log phase	1	120
X2	S4	log phase	5	60
X2	S5	log phase	5	120
X2	S6	overnight	0	0
X2	S7	overnight	1	120
X2	S8	overnight	5	120
X3	S1	log phase	0	0
X3	S2	log phase	5	15
X3	S3	log phase	5	30
X3	S4	log phase	5	60
X3	S5	log phase	5	120
X3	S6	overnight	0	0

*This genomic DNA sample was purchased from American Type Culture Collection (10).

Experiment X0 was sequenced in paired end mode, the others in single end mode. Experiments X0 and X1 were sequenced using MiSeq, and X2 and X3 using NextSeq. Each of the X2 samples were sequenced both with and without prior exonuclease treatment.

**Figure 2.**
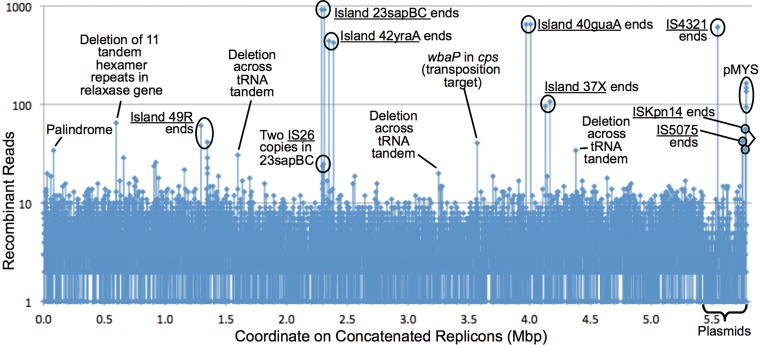
Juxtaposer program reveals DNA mobility events. Each Juxtaposer output read names two juxtaposed genomic coordinates. These coordinates, from all 22 543 output reads for the samples not treated with exonuclease, were counted and mapped to 500-bp genomic windows. The high-count coordinates identified the two ends of several mobile elements (underlined), palindromic PCR artifacts, counts due mainly to high copy number of one plasmid (pMYS, whose copy ratio to the chromosome core was 74.7 in the combined data from these samples), deletion scars for tandem repeats (also potential PCR artifacts) and a plausible target of positive selection (*wbaP* gene encoding the first enzyme in the capsule polysaccharide synthesis pathway, disrupted by three independent IS*Kpn14* transposition events). Counts for 23sapBC ends are exaggerated by one experiment in which the island was pre-deleted.

The primary method used here to examine DNA mobility with NGS data is the program Juxtaposer (Supplementary Figure S1). This discovery software finds reads whose left and right ends match non-adjacent genomic locations; such non-standard reads may identify short-range recombination events, such as transpositions, the circularization junction (CJ or *attP*) of a mobile element, or the chromosomal DJ (*attB*) left upon deletion of the element. Its main steps are listed in SI; the major filter removes standard reads that fully match a span of the standard genome. The output recombinant reads emphasize mobility events of GIs and ISs (Figure 2).

**Figure 3.**
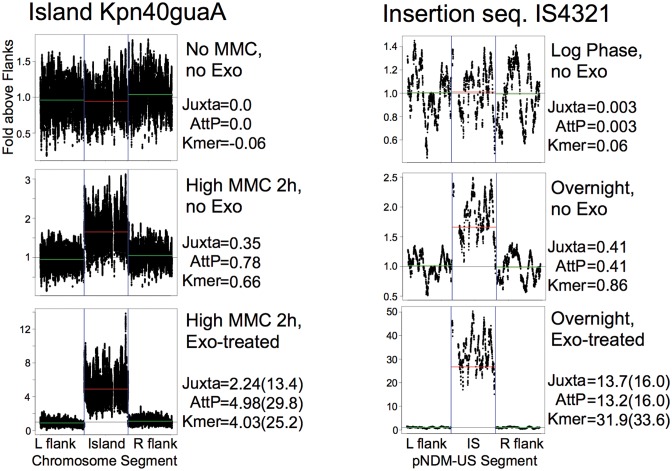
Methods. NGS analysis methods revealing mobility, for two mobile elements. The top panels are from samples with little mobility activity; values for the element are from Juxtaposer, AttCt and average unique 21-mers (Kmer), all normalized to **F** (average unique 21-mer count for 30-kbp flanks). The plots show (x-axis) positions along the genome (120 kbp centered at Kpn40guaA and 4 kbp centered at IS4321) and (y-axis) read counts for each genome-unique 21-mer normalized to the average for the flanks. The middle panels are from samples where the elements have mobilized; the excesses over the flanking sequence may be due to replication, either post-excision for the island or as part of the circularization mechanism for the IS. The bottom panels are from the exonuclease-treated sample partners of the middle panel samples, showing resistance of circular molecules; expected values for a similar sized plasmid are given in parentheses. For the genomic island (GI), the observed exonuclease resistance is significantly (∼6-fold) less than expected from plasmids.

Two additional methods were employed: (i) once mobile element ends were discovered and mapped, somewhat more sensitive tests for CJ and DJ reads could be devised, which were counted using our AttCt software. (ii) When levels of free excised copies of single-copy elements rose above the background from the original genomic copy, they could be measured using coverage by the unique k-mers of the whole element in comparison to its flanks. Figure 3 shows examples of results for two mobile elements that were highly active, the GI Kpn40GuaA and the insertion sequence IS*4321*. Each is shown for a low-activity and high-activity sample, and with an exonuclease treatment that shows resistance of the mobilized DNA, suggesting that it is at least partly in circular form.

### Genomic island CJs and DJs

Table 1 summarizes the Juxtaposer and AttCt results for combined experiments. For 6 of the 11 GIs, Juxtaposer identified numerous CJ reads and smaller numbers of DJ reads, and these counts increased with AttCt. The *att* site sequences validated by these reads, and potential effects on target gene products and expression, are shown for these islands in Supplementary Figure S2. By providing the recombinant *attP* and *attB* sequences, Juxtaposer output precisely maps island termini onto the genome. Coordinates for active islands that the Islander program (6) had mapped bioinformatically to tRNA and tmRNA genes were confirmed, while the new data altered the previously predicted coordinates of three Kpn2146 islands integrated into protein coding genes, in one case by over 3 kbp. These remappings validate the ability of Juxtaposer to discover new mobile DNAs.

**Figure 4.**
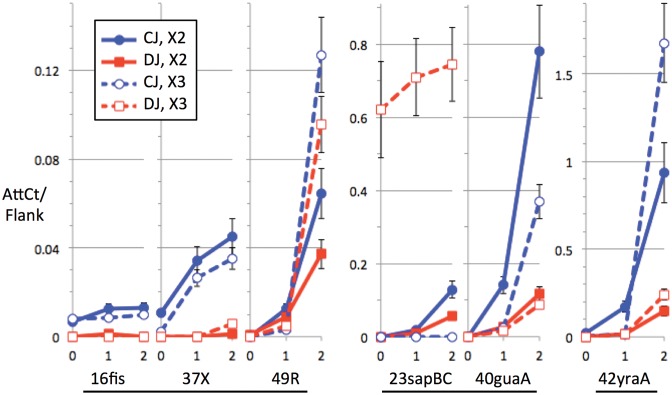
Timecourse. The AttCt timecourse data (normalized to the F measure of chromosomal flanks, see ‘Materials and Methods’ sections) for the high mitomycin dose in log phase (experiment X2:S1,S4,S5 and X3:S1,S4,S5) are shown for six islands, all suspected prophages. Three groups of graphs (underlined) use different y-axis scales, increasing to the right; y-axis is AttCt measures of CJ and DJ reads normalized to **F** (coverage of flanking sequence). CJs (blue) may be more numerous than DJs (red) due to post-excision island replication. Measurement error was propagated from that of **F** (standard error in unique 21-mer counts across the flanks).

Figure 4 shows two biological replicates of brief timecourses for the response to a high MMC dose, for the six active islands. Except for a gross difference between the two experiments (Kpn23sapB was apparently pre-deleted in experiment X3), trends were similar, with some differences in extent; Kpn49R and Kpn42yraA were somewhat more active in X3 than X2, with the contrary for Kpn40guaA. These results show differential biology among the islands in terms of extent of excision, onset of excision and CJ/DJ ratio. Excision alone should produce stoichiometric amounts of CJ and DJ. However the typical next step after excision would be theta mode replication of the circular island, at a faster rate than replication of the chromosomal DJ (5). Thus differences in CJ/DJ ratio may reflect different lags or rates of replication.

The circular nature of the molecules containing various island *att* forms was interrogated with an exonuclease that according to the manufacturer is active on linear dsDNA (less so on linear or circular ssDNA), but not on nicked, closed or supercoiled circular dsDNA. Circular DNAs survive extraction protocols dependent on their size, except for chromosome-sized circles which are so large that they virtually never purify intact. The size dependence of exonuclease resistance of DNA circles was internally calibrated using the four endogenous plasmids (which range from 2–141 kbp), separately for each sample (Supplementary Figure S3); even the largest plasmid was more resistant to exonuclease than the chromosome. Islands with CJ-forming activity were resistant to exonuclease, but not to the extent expected from the plasmid calibration curves (Supplementary Table S1). These findings are consistent with replication as modeled by phage lambda, which eventually produces linear genomic DNA production in rolling-circle mode, with the genome length cuts occurring at a site distant from the CJ/*attP* site (5).

### IS circles

**Figure 5.**
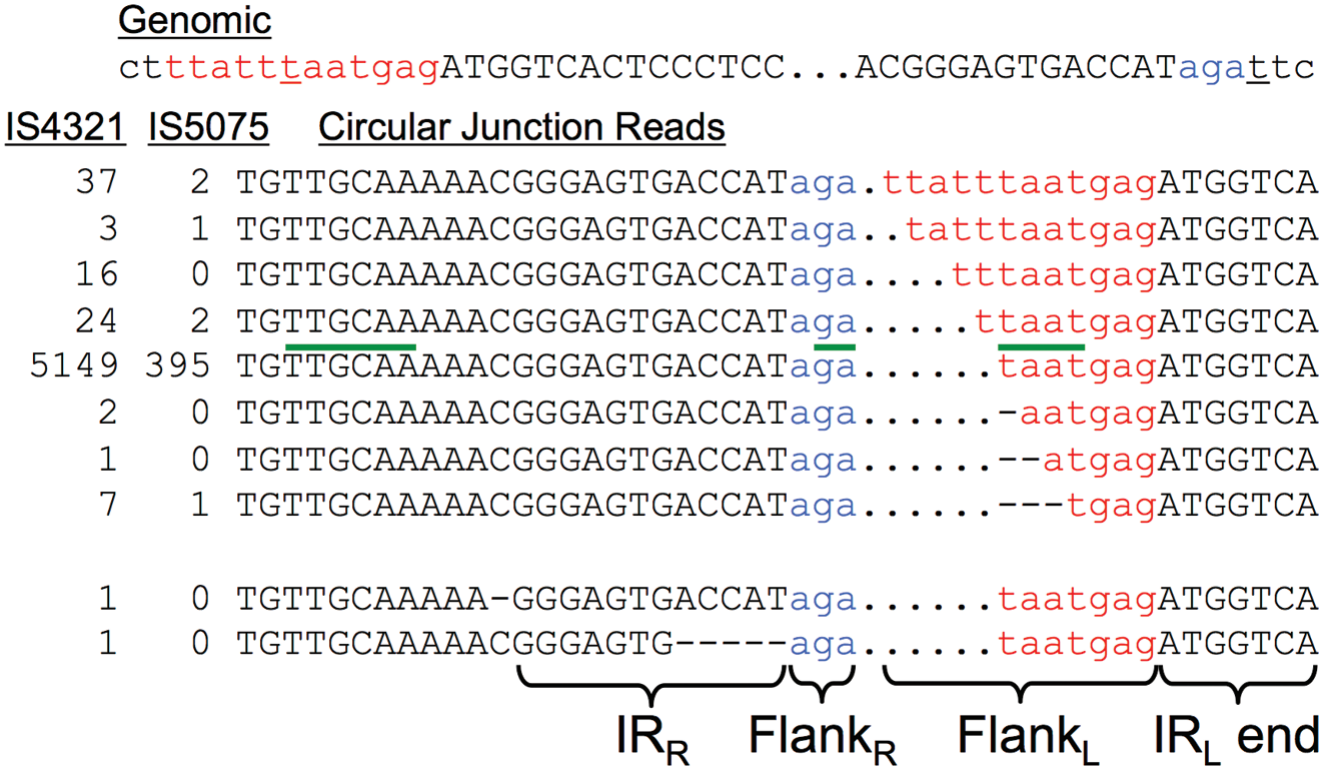
Left flank size fidelity in IS*4321* and IS*5075* CJs. Upper panel: genomic sequences at IS ends; red, left flank sequence; blue, right flank sequence. This sequence is the same for both ISs; they target the same conserved end of Tn*21*-like transposons (11). It had been unclear which underlined T was the source of a particular T present in the usual CJ sequence (11). Lower panel: read counts for CJ sequences, considering only indels (i.e. ignoring base substitutions). Green overline, putative −35 and −10 promoter boxes (11). The predominant sequence is the same as that described previously for CJs (11). The bottom two sequences were the only ones found with indels outside of the flank junction.

Two of the experiments compared overnight and log phase cultures. For unknown reasons, in one experiment (X2) but not the other (X3), the overnight culture produced a bonanza of CJs for seven types of ISs (Supplementary Figure S4 and Figure 5). Consistent with known transposition mechanisms, no DJs (from simple deletion of the original mobile DNA) were found for any of the ISs. The flanking donor ends that might be left immediately after cut-and-paste transposition would require more specialized (ligation-based) sequence library preparation methods for detection.

CJs join the left and right IS ends with a linker sequence from one or the other flank, allowing identification of the active copy among multi-copy ISs (Supplementary Figure S4). This demonstrates differential activity of IS copies: 90% of IS*Kpn18* CJs came from one of two copies (*P* = 4.2 × 10^−9^, binomial distribution), 67% of IS*Kpn21* CJs came from one of two copies (*P* = 1.2 × 10^−13^) and 72% of IS*26* CJs came from one of the 11 complete copies (*P* = 4.6 × 10^−14^). This strong differential activity is remarkable since the sequences of the IS copies are virtually identical.

The ISs show different regularity with respect to the linker length in CJs (Supplementary Figure S4). For ISKpn18, the linker length is always exactly 3 bp, agreeing with the 3-bp direct repeat (DR) found upon its transposition. For IS*Kpn21*, there is an experiment-to-experiment difference in the linker length, suggesting that its mechanism may depend on undetermined physiological variations; in experiment X0 it is 5 bp for all six instances, matching the DR length, while in Experiment X2, it is 1–3 bp for 440 reads. IS*26* has widely linker lengths, from either flank, that rarely match its 8-bp DR length. Some IS*26* CJ sequence lengths approach the detection limit of read size, suggesting they may reflect only the tail of a larger size distribution, as becomes clearer below.

The related ISs IS*4321* and IS*5075* are special in their site-specificity and their inclusion of canonical short additional sequences at each end, beyond the inverted repeat (11). These are the most rampantly circularizing ISs in these experiments, measured by AttCt at rates of 0.85 CJ/flank for IS*4321* and 0.079 for IS*5075* in the most productive exonuclease-untreated sample. The numerous CJ sequences allow fine examination of the imprecision of transposases as they produce CJs. Base substitutions that might arise during circularization would be difficult to distinguish from those arising during sequencing; instead indel errors are emphasized, as they are rare artifacts in Illumina sequencing (Figure 5). The CJ sequences provide an internal estimate of 8.6 × 10^−6^ for the indel sequencing error rate (the rate of indels at positions other than the flank junction). The indel rate is much higher (1.7 × 10^−2^) at the flank junction itself, and can be ascribed to extra or missing sequence from the left flank; these were assigned to transposase specificity errors. This error rate is still sufficiently low that the proposed −10 promoter box (11) would only rarely be affected. However the flank junction errors resolve mechanistic questions, such as the ambiguity concerning which flank is the source of the underlined T in the CJ sequence AGATAATGAG (11); the progression of error sequences, particularly those where the AGA right flank abuts a non-T from the left flank, suggests that the T in question comes from the *left* flank.

The model IS for circularization, IS*911*, precedes full circularization with a figure-eight form of the donor, in which only one IS strand is circular and the other is connected to its original donor flanks (3); it is not clear how this form might respond to the exonuclease used here. The CJ molecules of IS*4321* and IS*5075*, unlike those for GIs, respond to exonuclease approximately as predicted from the plasmid calibration curve (O/E values near 1 in Supplementary Table S1).

### Transpositions

The extensive IS circularization suggested that there may have been transposition events during bacterial culture that our methods would also reveal. After Juxtaposer marks reads as CJs, DJs or palindromes, it enables a subsequent search for transposition events among recombinant reads, by identifying those involving the terminus of a potential transposable element, in the appropriate configuration. A reference list of annotated Kpn2146 elements was prepared, comprising 59 transposable elements with at least one end intact, of 27 types and including 26 additional potential mobile DNAs of Kpn2146 (GIs, group II introns and integron cassettes) to allow detection of their promiscuous insertions. This identified 334 reads, which, combining cases of the same juxtaposition in the same experiment (that may have already been present in the culture prior to sample splitting), comprised a minimum of 251 potential transposition junctions. The average read count per transposition junction (1.33) is small, but fluctuations by experiment suggest that the junctions were clonal, arising at different times prior to harvest; only one appeared independently in two experiments. Most junctions (89%) came from three IS types: IS*26*, IS*Kpn14* and IS*Kpn18*.

Transposition rates were not proportional to circularization rates for IS types (Table 2), despite the opinion that circles are transposition intermediates (2,3,12). IS*4321* and IS*5075* should be excluded from this question, because their site-specificity and prior occupancy of those sites suffice to explain their non-transposition, as should IS*26* whose circles appear as a continuum with transposition events. After these exclusions, IS*Kpn1* and IS*Kpn21*, which produced little transposition relative to circles, can be contrasted with IS*Kpn14*, which behaved conversely.

#### Transposition events where both ends could be detected

Fourteen same-experiment pairs of transposition junctions could be matched as the putative left and right end of the same transposition event (Supplementary Figure S5). The three end-matched cases involving IS*26* exhibited the expected 8-bp DR, and the remaining 11 cases involving IS*Kpn14* showed 9-bp DRs (except for one case of an 8-bp DR); the DR length had not been previously determined for IS*Kpn14*, but is compatible with the 9-bp DR typically found for other members of the IS*1* family (13). One two-IS event apparently mobilized a large plasmid segment containing many resistance genes, with IS*Kpn14* at one end and a partial copy of IS*1X4* (also a member of the IS*1* family) at the other end.

Cases of IS polarity inversion were observed, where the IS gave the appearance of having flipped orientation in situ, both for IS*Kpn14* and for IS*26*. At a single new site in *cspC* both ends of both orientations of IS*Kpn14* were found in the same sample (Supplementary Figure S5).

#### Plasmid preferences

**Figure 6.**
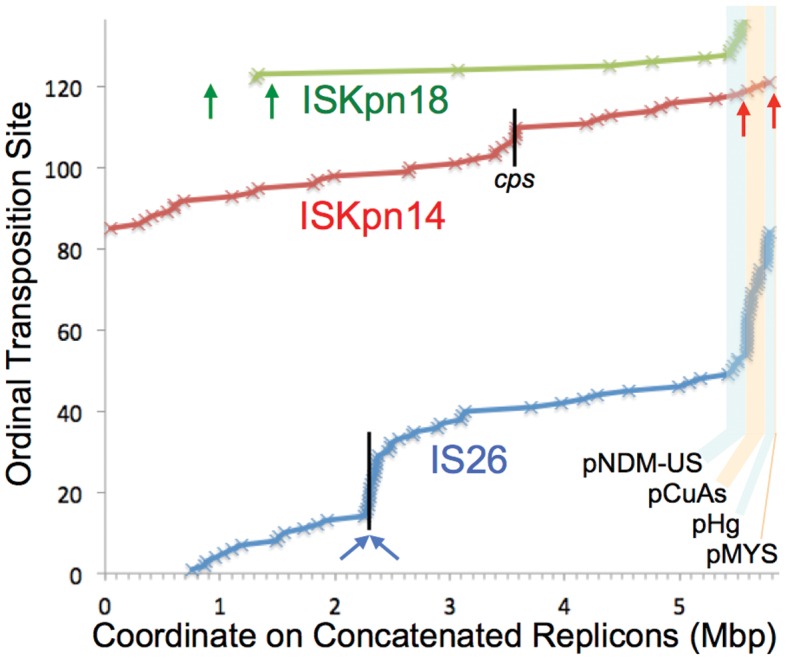
Hotspots. Transposition sites from exonuclease-untreated samples were ordered by the top three active IS types and then by coordinate on the concatenated replicons. Arrows mark the native copies of each of these IS families (omitting here the numerous IS*26* copies on pCuAs and pHg). Black vertical lines mark chromosomal sites showing significant transposition site clustering (SI, ‘Statistics of transposition site bias’), at the capsular polysaccharide synthesis locus *cps* for IS*Kpn14* and at the two native chromosomal copies of IS*26*.

Transposition sites from exonuclease-untreated samples were mapped along the genome, revealing IS specific replicon and regional preferences (Figure 6). Replicon preference was evaluated statistically (Table 4), showing that each of the main active IS types is over-represented in at least one of the four plasmids, in some cases with corresponding under-representation in the chromosome. IS*Kpn18* shows extreme preference for the conjugative type 1 IncA/C_2_ plasmid pNDM-US. IS*Kpn14* transposition is over-represented in pCuAs. IS*26* transposition is enriched in the two plasmids where it was originally found (pCuAs and pHg), part of the local action phenomenon described below, but was also enriched in pNDM-US where it was not already present.

**Table 4.** Enrichment factors for transpositions into replicons

	Replicon				
	Chromosome	pCuAs	pHg	pMYS	pNDM-US
IS*26*	0.10*	35.87^†^	13.71^†^	0.38	4.56^†^
IS*Kpn14*	0.91	4.90^†^	0	0.44	1.01
IS*Kpn18*	0.04*	0	0	0	119.51^†^

*Significant under-enrichment (*P* < 0.01).

^†^Significant over-enrichment (*P* < 0.01).

Data from the samples not treated with exonuclease were combined.

#### Transposition site clustering

Some IS types on some replicons showed little clustering of transposition sites; notably, IS*Kpn18* sites were randomly distributed on pNDM-US. In other cases there was significant clustering. IS*26* showed local action, preferring the region of its only two native chromosomal copies, and pCuAs and pHg, where IS*26* is also native. This local action, together with the widely varying IS*26* CJs (Supplementary Figure S4), suggests that the IS*26* CJs and transpositions represent the same intergraded phenomenon (i.e. the CJs are simply shorter-range local transpositions), thus in the subsequent section IS*26* CJ and transposition data are treated together. For IS*Kpn14* and IS*Kpn18*, whose transpositions do not cluster at their native sites and whose CJs have more discrete flank sizes, we continue to treat transposition as a separate phenomenon from circularization.

IS*Kpn14* on the chromosome was enriched in the *cps* region, the highly varied multi-gene locus of capsular polysaccharide synthesis (10). The four *cps* sites were especially significant considering that they occurred only in the two smallest (MiSeq) experiments, which together had yielded a total of only 20 transposition sites for the three main IS types combined. These *cps* disruptions include the most highly expanded clones for any transposition event in any of the experiments, reaching 25 read counts and sufficient to detect both transposition junctions in two cases (Supplementary Figure S5). Three of the *cps* transpositions independently disrupted a single gene *wbaP* whose product catalyzes the first step of O-antigen synthesis, and the fourth disrupted a glycosyl transferase gene. Together these observations suggest that there was selection against capsule production during the two smaller experiments.

To more fairly compare replicon preferences, Figure 6 had excluded data from the plasmid-biased exonuclease treatment. However to examine potential IS hotspots *within* an individual replicon, signals were boosted by pooling all data (Supplementary Figure S6). The extra data did not alter replicon preference for IS*Kpn18*; it still preferred only NDM-US, yet with no particular hotspot. This pattern suggests that IS*Kpn18* may recognize a unique but dispersed ‘attractant’ feature unique to this 141-kbp replicon. One candidate for such a feature is the pNDM-US-encoded conjugation gene activator AcaCD (14); the *P*-value threshold 10^−6.4^ for its predicted binding sites in the Kpn2146 genome leaves 16 sites, all on pNDM-US. For nine of the ten IS*Kpn18* transpositions into pNDM-US, the average closest distance to one of the 16 AcaCD sites is 1780 bp.

For IS*Kpn14*, exonuclease treatment effectively shifted most detected transpositions from the chromosome to the cryptic ColE1-family plasmid pMYS (Supplementary Figure S6). This can be understood primarily as an effect of massive enrichment of tiny pMYS; indeed it is so enriched by exonuclease as to account for 74% of all reads, compared to 2.4% in exonuclease-untreated samples. Overlaid on this shift to pMYS is a preference for a particular region within it. Only part of this regional preference may be explained by known vital plasmid regions; i.e. pMYS bearing an IS in the 555-bp region of the plasmid that encodes the primer RNA of replication should fail to replicate. It is notable that all these transpositions map within or just upstream of the largest pMYS ORF. Although this ORF has no detectable homology with the copy control gene *rop* found in other ColE1 family members, if it does have a similar function, these transpositions should yield even higher pMYS copy numbers, which might explain the apparent enrichment here.

### IS26 local activity

To unify all relevant IS*26* data (CJs, transpositions, and other potential phenomena), all recombinant reads from Juxtaposer were collected in which one hit mapped within 10 bp of any IS*26* copy end. Native Kpn2146 IS*26* copies are found on the chromosome (copies 1–2), pCuAs (copies 3–5) and pHg (copies 6–12); they have identical terminal sequences, except that copy 8 is partial and copy 5 has a single base substitution 31 bp internally from its right end, marking this copy uniquely. Some transposition sites were difficult to map to the genome because they fell in repetitive DNA (Supplementary Figure S7), including transpositions into internal sites of IS*26* itself, although some of these sites could be nonetheless be disambiguated. Fusions of IS*26*-flanking sequence directly to a new target site, as might occur if IS*26* transposase ever behaved like a tyrosine or serine recombinase, could have been detected, but were not.

For samples unbiased by exonuclease treatment, transposition sites that mapped unambiguously to the chromosome were spread along it, but not evenly, concentrating at the region of the two native copies of IS*26* (Figure 6). A recent study of IS*26* emphasizes that replicative transposition within the same replicon yields either an inversion of a flanking segment or deletion of a flanking segment concomitant with its circularization (15). The local concentration of transposition junctions was observed for all four configurations (Supplementary Figure S8A), i.e. left (L) or right (R) IS*26* end, aiming upstream (+) or downstream (−). Detailed interpretation of this pattern is somewhat confused by the presence here of two nearby native IS*26* copies, in opposite orientations (Supplementary Figure S8C). It is clear however that balance between inversions and circularization/deletions, due to local action of even just one of the two copies, should produce a balance of the four configurations throughout the region, as observed (Supplementary Figures S8C and 9).

Circular products of local IS*26* action should be resistant to exonuclease treatment, but less so for inversion and deletion products. Its effect was to significantly increase the local concentration, indicating that IS*26* circles do indeed arise from this and only this region (Supplementary Figure S8B). Moreover, the configurations were mainly restricted to two types, L+ and R−, those expected for circles of IS*26* copy 1; closeups of the mappings confirm that they are consistent with circular forms of copy 1, with several L+ configurations on its right side and R− configurations on its left side (Supplementary Figure S8D). These circles include the CJ reads described above, and circles whose junction would be too long to detect in short reads, i.e. a spectrum of linker lengths. Longer circles may also be produced, but would be less favored by size dependent exonuclease resistance (Supplementary Figure S3). The neighboring, opposite-orientation IS*26* copy 2 shows much less activity, although some of its circles are indicated by L− at its right and R+ at its left. The two IS*26* copies surround a 4.3-kbp segment containing an integron fragment with an intact *aadA2* streptomycin resistance gene. These copies lie within the active island Kpn23sapBC, which itself is amplified relative to the surrounding chromosome. However measuring levels of each island and the remaining chromosome core (using unique 21-mers) shows that amplification of this island target is not sufficient to account for the clustering of IS*26* transpositions here, with or without exonuclease treatment.

Of the 13 IS*26* transpositions into IS*26*-internal sites (Supplementary Figure S7A), 12 were from exonuclease-treated samples, suggesting they are circular forms. Moreover 11 showed configurations consistent with circles (L− or R+). These may be considered the extreme of the spectrum of local IS*26* activity. Neither the end nor the internal site could be mapped to any particular IS*26* copy, with one exception, where both could be mapped; this case represented an attack at a non-self copy and is therefore also an exception to local activity.

There were also many IS*26* transposition events on the IS*26*-bearing plasmids pCuAs and pHg (Supplementary Figures S10 and 11). Exonuclease resistance does highlight IS*26* circles, but not as sharply as for the chromosome, perhaps because the plasmids themselves are also somewhat resistant. On pCuAs the tandem copies IS*26*.3 and IS*26*.4 show more local activity than the third nearby oppositely-oriented copy. On pHg the highest concentration of exonuclease-untreated events maps far from any IS*26* copies, seemingly contrary to the local activity rule.

IS*26* copy 6 on pHg has a single base substitution 31 bp internally from its right end, marking this copy uniquely. In the one CJ read whose linker sequence identifies its source as the left end of IS*26*.6, the right end is the marked one from IS*26*.6, showing that a single IS*26* copy provided both ends of a CJ. Of the 13 events involving the IS*26*.6 right end, 11 fall in its own replicon pHg; the other two fall in the main sink, the chromosome, but not in its hotspot region. This suggests that copy-local activity reflects self-activity more than an attraction of external copies.

Polarity inversion was observed at native IS*26* copies with new left ends swapping for the native right ends, specifically detected at native IS*26* copies 3, 10, 11 and the partial copy 8; occurrence at the partial copy suggests that the phenomenon is not simple inversion of an existing IS but begins with attack by a remote IS copy. Polarity inversion was also observed with newly transposed IS*26* copies, at pCuAs/11932 and at pCuAs/11134.

## DISCUSSION

Classically, microbiologists worked with phage and bacterial stocks and thought of integration as the forward reaction for integrase. Now, ready access to numerous genome sequences inspires testing for excision products, to discover or verify new integrase site specificities. Our approach globally examines such events, providing unbiased experimental avenues to island discovery that complement existing bioinformatic approaches to island identification. It can also be applied to the study of transposition, here suggesting several principles for biased transposition distributions or hotspots, including positive selection, local activity, regional preferences and replicon preferences. Future modifications will allow measurement of the phases for the invertible DNAs that promote phase variation.

Although the duration of these experiments was brief, there was sufficient time for an unintended selective pressure, against capsule synthesis, to act detectably. While the characteristic thick capsule of *Klebsiella* is essential for virulence and important in the organization of biofilms, acapsular mutants accumulate spontaneously during continuous *in vitro* culture (16). This has also been demonstrated in *Streptococcus pneumococcus* where 85% of such mutants had point mutations in *cpsE* (17,18), a homolog of the *Klebsiella wbaP* gene that accumulated multiple ISKpn14 transpositions here. At another extreme of selective pressure, the transposition events observed need not be stable over extended culture; they may be unstable, even lethal, mutations detected before elimination. As an example, one IS*26* event would have truncated by 50% the single-copy glycolysis gene *pgk* which is essential in *Escherichia coli* (19).

Only short reads were analyzed here, which can detect recombination over short crossover segments, as is catalyzed by site-specific recombinases and transposases. The typical dependence of homologous recombination on longer homologies (dramatic declines in efficiencies for homology < 75 bp (20)) disfavors its detection within short read sequences. Long, PCR-free reads can instead detect recombination across rRNA operons and other long repeats (10). Intramolecular replicative transposition produces rearrangements (circles, inversions and deletions) as it duplicates the transposable element (15); our main method is blind to these events because their detection requires longer-range information, i.e. read lengths longer than the element length (although circularity was addressed here independently through the use of exonuclease). Likewise the TU circles described for tandem IS*26* pairs, produced by the transposase through both replicative and non-replicative mechanisms, yield no new DNA sequence juxtapositions (Supplementary Figure S9) and would require other methods to detect (4).

Other Kpn2146 plasmids may be mobilizable, but only pNDM-US encodes its own conjugation pilus. IS*Kpn18* transposed with unexpected frequency into pNDM-US, yet with random distribution around the plasmid. This may indicate a mechanism with evolutionary implications, through which this IS detects and boards a plasmid about to transfer to another bacterial cell. Possible plasmid-marking features could be (i) the complex at the cytoplasmic platform of the conjugation pilus as the plasmid moves, (ii) a factor loaded onto only its replisome or (iii) a unique evenly-spaced plasmid-binding protein. As a possible example of the latter, transpositions were close to the binding sites for the conjugation gene transcription activator AcaCD. Regarding replisome tracking, several transposases have been found to interact with the host beta sliding clamp (21); it will be interesting to learn whether any beta clamp or other factor specifically marks the replisome on this plasmid.

IS*26* is most commonly found on plasmids, and known for mobilizing flanking antibiotic genes among enterobacteria (4). Although our data show some preference of IS*26* for targeting plasmids, local activity can be substantial even in the chromosome. Local transpositions were not inherently biased toward circle-forming configurations, although exonuclease treatment enriched for these. The preference of IS*26* for local action should primarily produce rearrangements, deletions and disruptions of local genes (15), which would more likely be tolerated in plasmids than in chromosomes.

We focused on the recombination events involving the ends of mobile DNAs, but a larger number of unexplained recombination events are detected by Juxtaposer. They may be partly or mostly due to artifactual PCR-mediated recombination, including palindrome formation, occurring during library preparation; PCR-free library preparation methods should help to resolve this. Alternatively, they may represent a pervasive recombination phenomenon, real non-homologous recombination events that occur with little site specificity. Any such low-level phenomena do not alter our primary results, which stand well above this background (Figure 2).

Our approach has some of the same goals as comparative genomics, yet it provides much richer information than can be gleaned from a handful of arbitrarily sampled frozen accidents. It is similar to other recent softwares that look for short reads that support mobility events, breseq and ISMapper [refs], but these treat the sample as having a single uniform genome that may differ from a reference, while Juxtaposer searches for subpopulations within the sample that have undergone different mobility events, and also explicitly searches for CJs. Our approach provides the control aspect of working within a single genetic background, and it can be applied to organisms that may have few related genomes available. It is richly comparative when applied to a rich mobilome, allowing comparison of the behaviors of multiple mobile DNAs at once, or comparison of different physiological settings on mobility mechanisms. In current work, Juxtaposer is providing new insight into the *Clostridium difficile* mobilome. We recommend routinely including a side MMC-treated culture in bacterial genome sequencing projects, to enable discovery and annotation of GIs.

## ACCESSION NUMBERS

CP006659, CP006660, CP006661, CP006662 and CP006663.

## Supplementary Material

SUPPLEMENTARY DATA
