# Supplementary material for: Experimental single-strain mobilomics reveals events that shape pathogen emergence
Source: Nucleic Acids Res. 2016 Jul 4;44(14):6830–9. doi: 10.1093/nar/gkw601 (PMC5001619; doi:10.1093/nar/gkw601)
Supplement: SUPPLEMENTARY DATA [file supp_44_14_6830__index.html]

Experimental single-strain mobilomics reveals events that shape pathogen emergence — Experimental single-strain mobilomics reveals events that shape pathogen emergence — SUPPLEMENTARY DATA 

# Experimental single-strain mobilomics reveals events that shape pathogen emergence

## SUPPLEMENTARY DATA

- SUPPLEMENTARY DATA
- SUPPLEMENTARY DATA
