# Supplementary material for: Experimental single-strain mobilomics reveals events that shape pathogen emergence
Source: Nucleic Acids Res. 2016 Jul 4;44(14):6830–9. doi: 10.1093/nar/gkw601 (PMC5001619; doi:10.1093/nar/gkw601)

**SUPPLEMENTARY INFORMATION** for **“**Experimental single-strain mobilomics reveals events that shape pathogen emergence” by Joseph S. Schoeniger, Corey M. Hudson, Zachary W. Bent, Anupama Sinha, and Kelly P. Williams

*Juxtaposer software:* Juxtaposer was developed to find high-throughput reads corresponding to the short-range recombination events of DNA mobility (Fig. 1 and Fig. S1). Paired end reads should be merged first; Juxtaposer does not explicitly consider pairs (we have been concerned with barcode bleed artifacts). The following initial filters are applied to yield recombinant reads:

i) A quality filter trims low-quality segments and primer sequences ([1](#_ENREF_1)).

ii) In the most effective filter (~0.5 % pass rate), standard reads are identified for rejection, using Bowtie2 ([2](#_ENREF_2)) in global, sensitive mode against the standard genome sequence. The index includes an entry for the closure sequence of each circular replicon (i.e., one read length of sequence from each flank of the circular origin).

iii) Nonstandard reads are queried against the genome and its closures using BLASTN with a 95 % identity filter, rejecting reads that yield exactly one or zero hits.

iv) Multi-hit reads are tested for the shifted configuration (as opposed to the nested configuration). The top-scoring hit (or hits, if tied) is tested pairwise against all other hits for the read, keeping reads only if a tested hit pair each had at least eight bp unshared distally. Two shifted hits on a read have an overlap that can be negative (a gap where neither matches) or zero (an abutment). If multiple shifted hit pairs were found, the closest on the same replicon are reported.

The output from each recombinant read is the pair of genomic coordinates (a juxtaposition) from the overlap endpoints of the pair. Juxtaposer reports unique juxtapositions along with the read count (and a sample read) supporting each one. Juxtapositions are labeled as potential circular junctions (CJs), deletion junctions (DJs), or palindromic artifacts ([3](#_ENREF_3)) when shifted hit pair coordinates are from the same replicon. If the hits have the same orientation, the juxtaposition is either labeled CJ (if the hit order is reversed relative to the replicon) or DJ (if the hit order is in the same as in the replicon). If same-replicon hits are oppositely oriented and separated by < 10 kbp the juxtaposition is labeled a palindrome.

To characterize mobile elements that might be responsible for juxtapositions, Juxtaposer includes software to identify and annotate genes for the enzymes promoting mobility of mobile elements, from a raw assembled reference genome. This includes Prodigal ([4](#_ENREF_4)) for gene identification and rFind ([5](#_ENREF_5)) for RNA identification. Transposons are identified using 96 hidden Markov models (HMMs) from TpnPred ([6](#_ENREF_6)). Integron integrases were collected using ACLAME FamInt8 ([7](#_ENREF_7)). Additionally, we include additional Pfam transposase (PF04754.7, PF14319.1, PF1490.1), reverse transcriptase (PF000078.22), recombinase (PF07508.8, PF13408.1), homing endonuclease (PF05203.11) and resolvase (PF00239.16) HMMs. Genes containing these domains are annotated in a mobility gene file. Genomic islands are identified using Islander ([5](#_ENREF_5)). IS coordinates can be identified with help from ISfinder ([8](#_ENREF_8)). Additional islands, ISs or other mobile elements may be annotated from CJs/DJs discovered in preliminary Juxtaposer runs.

Juxtaposer uses mobile element or mobility gene information to further label juxtapositions. CJs, DJs and palindromes are labeled with the included mobility gene (if any) closest to one of the coordinates. A fourth juxtaposition category, transposition, is triggered when one coordinate of a shifted hit pair is located within a short distance from the end of an annotated mobile element.

Juxtaposer has a BSD-2 clause license. It runs on Unix/Linux systems with dependency on Perl, BLAST+ 2.2.27+, Bowtie 2.2.4, HMMER 3.0, Prodigal 2.6.1, and pfscan 2.3.

*Exonuclease treatment:* Circle-breaking damage can occur during genomic DNA preparation and cause exonuclease susceptibility; this damage depends strongly on circle size and may vary between samples. Expected resistance factors for circular GIs and ISs were calculated using plasmids as standards, as follows. Reads were assigned to each replicon using Bowtie2 ([2](#_ENREF_2)), masking the regions of the eight most active GIs and ISs. Raw read counts were boosted proportionally to the size of the masked segments, and each was normalized to the total assigned reads for the sample. Normalized replicon read counts for exonuclease treated samples were divided by those for their untreated partner samples, producing the enrichment factors plotted in Fig. S3. Predicted size-based, per-sample exonuclease enrichment factors for GI and IS circles were interpolated or extrapolated using just the two most relevant plasmids sizes (pHg at 85161 bp and pMYS at 2014 bp).

Observed values for post-excision GI and IS exonuclease enrichment factors were taken from attCt CJ counts, when counts in both the treated and untreated sample were ≥ 10. CJ counts were normalized to total assigned reads for the sample, and the value for the treated sample was divided by that for the untreated partner. Observed:expected ratios reported in Table S1 indicate the fraction of molecules that were circular in vivo, relative to plasmids that are considered virtually fully circular in vivo.

*Genome reassembly:* The initial Juxtaposer output pointed to several local errors in the previous assembly, mostly internal to the original Newbler contigs. For each, the raw sequencing data was re-checked using readStepper ([9](#_ENREF_9)), leading to corrections at the following seven sites: between *fhuA_2* and *ssuB_3* (119 bp inserted), the tRNA-Tyr/RtT region, the end of Kpn55F (163 bp inserted adding a second small undamaged attR), *lyx_2* (25 bp deleted restoring the reading frame, downstream of *livJ_2* (124 bp inserted revealing a second ERIC repeat), the two tRNA-Ser/RtT units, and the quintuple repeat of a DUF3304 gene (Kpn2146_2409-2413). The corrected genome was freshly annotated using Prokka ([10](#_ENREF_10)), retaining annotations that had previously been manually curated, and all mobile elements discussed here were added. This annotation was compared to the corresponding original RAST annotation, and non-hypothetical RAST calls missed by Prokka were retained. The revised genome project has been submitted to GenBank, as the second version of each replicon (accessions CP006659-CP006663).

*Statistics of transposition site bias*: We analyzed the statistics of the distribution of unique insertion sites (i.e., counting multiple reads for the same site from the same experiment as a single event) targeted by each IS. Three types of analysis were performed.

First the relative preference of an IS for different whole replicons was determined. The number of reads representing each replicon was taken for each sample or group of samples by counting unique 21-mers and allocating reads proportionately. For each replicon and for each IS type, the ratio was taken of unique transposition sites to the replicon read count (on-replicon rate), and the same ratio was taken for the other replicons combined (off-replicon rate). The ratio of on:off replicon rates was taken as the enrichment factor for transpositions into the replicon. Significance was evaluated by jointly modeling the on- and off-replicon rates as a Poisson process using the *Mathematica* (Wolfram Research) function GeneralizedLinearModelFit; a p-value was calculated using a chi-squared distribution with one degree of freedom.

Second, for each IS the uniformity of distribution of all insertion sites within a given replicon was tested using the *Mathematica* function DistributionFitTest against a uniform distribution, calculating p-values using a Kolmogorov-Smirnov test.

Finally, for non-uniform distributions, the degree of localization of the insertion sites was determined. The smallest window in which the number of insertion sites is enriched, is inevitably a window with an insertion site at either end. The localization statistical technique is to test for a replicon of ***N*** bases with a total number of insertions ***M***, the probability, based on a uniform distribution, of observing an interval of length ***d*** bracketed by insertion sites and containing ***m*** total insertion sites). There are Binomial**(*d***-2; ***m***-2**)** configurations for such a cluster times Binomial**(*N-d***; ***M-m*)** configurations for the excluded interval, out of Binomial**(*N***; ***M*)** states, to give (with normalization constants): (***M***-1) Binomial**(*d***-2; ***m***-2**)** Binomial**(*N-d***; ***M-m*) / ( (N-1)** Binomial**(*N***; ***M*) )** which is a hypergeometric function that can be calculated over the lengths ***d***=***m*** to ***d***=***N***-***M***+***m***, and cluster sizes from ***m***=2 on up. To calculate p-values, values of this function were tabulated and progressively summed to create a cumulative distribution function. P-values were then Bonferroni adjusted by the total number of observations (possible clusters) ***M*.**

*Correlation of IS*Kpn18 *transpositions with AcaCD binding sites:* The sequence alignment of the 17 major AcaCD binding sites of IncA/C plasmid pVCR94 was taken from Carraro et al ([11](#_ENREF_11)). A profile was prepared for the pVCR94 sites and applied to the Kpn2146 genome using MEME and MAST at the MEME Suite website ([12](#_ENREF_12)). A cutoff for the motif was found (p = 10^-6.4^) which left 16 sites in Kpn2146, all in pNDM-US. At a less stringent cutoff (p = 10^-5.1^) two additional sites appeared in pNDM-US but with 58 sites in other replicons. No sites were found within IS*Kpn18* down to the least stringent cutoff evaluated (p = 10^-4^, yielding 765 hits genome-wide).

Ten of the 16 IS*Kpn18* transposition junctions detected by Juxtaposer were in 141-kbp pNDM-US (p < 10^-5^, chi-squared). Distance to the closest of the top 16 AcaCD binding sites was taken, and omitting one outlier, the mean was 1780 bp. The outlier was close to one of the lower-stringency AcaCD sites.

**REFERENCES**

1. Kim, H., Jebrail, M.J., Sinha, A., Bent, Z.W., Solberg, O.D., Williams, K.P., Langevin, S.A., Renzi, R.F., Van De Vreugde, J.L. and Meagher, R.J. (2013) A microfluidic DNA library preparation platform for next-generation sequencing. *PLoS One*, **8**, e68988.

2. Langmead, B. and Salzberg, S.L. (2012) Fast gapped-read alignment with Bowtie 2. *Nat Methods*, **9**, 357-359.

3. Star, B., Nederbragt, A.J., Hansen, M.H., Skage, M., Gilfillan, G.D., Bradbury, I.R., Pampoulie, C., Stenseth, N.C., Jakobsen, K.S. and Jentoft, S. (2014) Palindromic sequence artifacts generated during next generation sequencing library preparation from historic and ancient DNA. *PloS One*, **9**, e89676.

4. Hyatt, D., Chen, G.-L., LoCascio, P.F., Land, M.L., Larimer, F.W. and Hauser, L.J. (2010) Prodigal: prokaryotic gene recognition and translation initiation site identification. *BMC Bioinformatics*, **11**, 1.

5. Hudson, C.M., Lau, B.Y. and Williams, K.P. (2015) Islander: a database of precisely mapped genomic islands in tRNA and tmRNA genes. *Nucleic Acids Res*, **43**, D48-D53.

6. Riadi, G., Medina-Moenne, C. and Holmes, D.S. (2012) TnpPred: A web service for the robust prediction of prokaryotic transposases. *Comp Funct Genomics*, **2012**.

7. Van Houdt, R., Leplae, R., Lima-Mendez, G., Mergeay, M. and Toussaint, A. (2012) Towards a more accurate annotation of tyrosine-based site-specific recombinases in bacterial genomes. *Mob DNA*, **3**, 1.

8. Siguier, P., Pérochon, J., Lestrade, L., Mahillon, J. and Chandler, M. (2006) ISfinder: the reference centre for bacterial insertion sequences. *Nucleic Acids Res*, **34**, D32-D36.

9. Hudson, C.M., Bent, Z.W., Meagher, R.J. and Williams, K.P. (2014) Resistance determinants and mobile genetic elements of an NDM-1-encoding *Klebsiella pneumoniae* strain. *PloS ONE*, **9**, e99209.

10. Seemann, T. (2014) Prokka: rapid prokaryotic genome annotation. *Bioinformatics*, **30**, 2068-2069.

11. Carraro, N., Matteau, D., Luo, P., Rodrigue, S. and Burrus, V. (2014) The master activator of IncA/C conjugative plasmids stimulates genomic islands and multidrug resistance dissemination. *PLoS Genet*, **10**, e1004714.

12. Bailey, T.L., Boden, M., Buske, F.A., Frith, M., Grant, C.E., Clementi, L., Ren, J., Li, W.W. and Noble, W.S. (2009) MEME SUITE: tools for motif discovery and searching. *Nucleic Acids Res*, W202-208.

13. He, S., Hickman, A.B., Varani, A.M., Siguier, P., Chandler, M., Dekker, J.P. and Dyda, F. (2015) Insertion sequence IS26 reorganizes plasmids in clinically isolated multidrug-resistant bacteria by replicative transposition. *MBio*, **6**, e00762-00715.

**SUPPLEMENTARY TABLE**

TABLE S1. Exonuclease resistance of post-excision GIs and ISs relative to plasmids. Observed:expected ratios for exonuclease protection, from the untreated and exonuclease-treated sequence datasets taken for each sample of Experiment X2. Observed values were AttCt CJ counts normalized to total assigned reads. Expected values were size-based, from the plasmid calibration curves of Fig. S3. Values are considered not determined (nd) unless *attP* counts were ≥ 10 for both the untreated and exonuclease-treated samples. Values for ISs are higher than any for the GIs.

| Sample | | Growth Phase | | MMC (μg/ml) | | Harvest (hr) | Genomic Island | | | | | | Insertion Sequence | |
| --- | --- | --- | --- | --- | --- | --- | --- | --- | --- | --- | --- | --- | --- | --- |
|  |  |  |  |  |  |  | 49R | 42yraA | 40guaA | 37X | 23sapBC | 16fis | IS*4321* | IS*5075* |
| S1 | log phase | | 0 | | 0 | | nd | nd | nd | 0.086 | nd | nd | nd | nd |
| S2 | log phase | | 1 | | 1 | | nd | nd | nd | 0.155 | nd | 0.149 | nd | nd |
| S3 | log phase | | 1 | | 2 | | nd | 0.068 | nd | 0.433 | nd | 0.318 | nd | nd |
| S4 | log phase | | 5 | | 1 | | nd | 0.034 | 0.009 | 0.059 | 0.179 | 0.091 | nd | nd |
| S5 | log phase | | 5 | | 2 | | 0.088 | 0.060 | 0.121 | 0.217 | 0.224 | 0.129 | 0.536 | nd |
| S6 | overnight | | 0 | | 0 | | nd | 0.061 | nd | nd | nd | nd | 0.626 | 0.785 |
| S7 | overnight | | 1 | | 2 | | nd | 0.050 | nd | nd | nd | 0.226 | 0.745 | 0.604 |
| S8 | overnight | | 5 | | 2 | | nd | nd | nd | nd | nd | nd | 0.950 | 0.891 |

**SUPPLEMENTARY FIGURES**

Fig. S1. Juxtaposer steps and their yields.


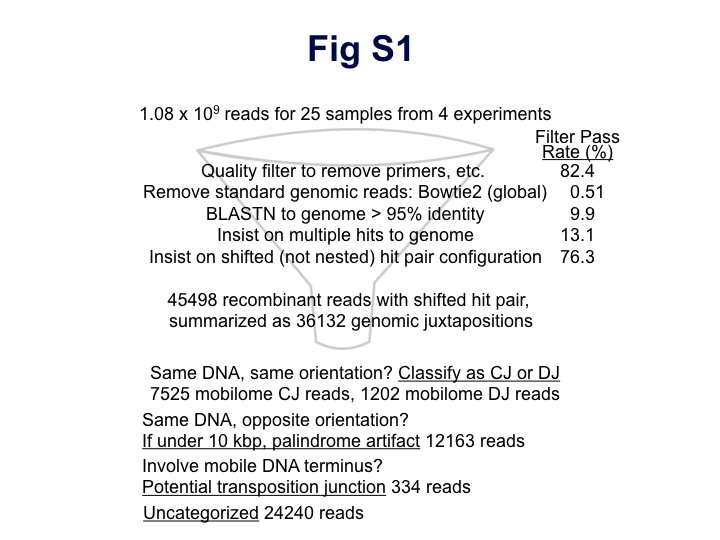


Fig. S2. Mapped island termini and effects of insertion on target genes. The genomic configurations of left and right ends of six genomic islands (red) and flanking chromosome (blue) are shown with possible effects on expression of target genes, twice for 23sapBC to show effects at *sapB* or at *sapC*. Underlines show the identity blocks within which crossover occurs as determined experimentally by Juxtaposer. Upper case: codons, and predicted (BPROM) ‑10 and ‑35 promoter blocks. Green over-lines mark nucleotides deleted in some Juxtaposer reads.

42yraA

2388204 TCGTCGgcatcctgaacggttcgCATTCTgcaagtcgccaggctatatctaagacacctttcgcccacaacgagacacaaag

fM S K K I L M L V G D Y A E D Y L N K I I T N *

2388122 ATGAGCAAGAAAATCCTAATGCTGGTCGGCGATTATGCCGAAGATTACTTGAACAAAATTATAACTAACTGA

upper case: promoter (BPROM score 4.27)

2345766 TATACAtaaacatctaaccgtattCATTCTtgtctgaacaatctcaaccat

fM Q S P H L A E D Y E T M V P F Q A L Q M …

2345715 GTGCAAAGCCCACACCTTGCCGAAGATTACGAAACGATGGTTCCTTTTCAGGCACTGCAGATG…

49R (tRNA-Arg)

1296774 GCGCCCTTAGCTCAGCTGGATAGAGCAACGGCCTTCTAAGCCGTAGGTCACAGGTTCGAATCCTGTAGGGCGTGCCAttaagaaacaa

1345908 tgattacagtgtgttatttaacgctctactttCTTCTAAGCCGTAGGTCACAGGTTCGAATCCTGTAGGGCGTGCCAtttaataatca

37X (tmRNA)

4129479 …GTAAAGACTGACTAAGCATGTAGTGCCGAGGATGTAGGAATTTCGGACGCGGGTTCAACTCCCGCCAGCTCCACCAaataaaacaag

4166045 taaatatctgttttcattacacaacaaatggtATGTAGGAATTTCGGACGCGGGTTCAACTCCCGCCAGCTCCACCAaaattctccat

23sapBC (showing *sapB* CDS)

…L G A M A N P L K H K E W Y A L R *

2309782 …CTCGGTGCCATGGCTAACCCACTGAAACATAAGGAATGGTATGCCCTACGATAGcacctacaaaataattatttttg

R *

2286483 aaagacttaaagcatgactaacaaacttaaaacggattcatatgcccTACGATAGcgtttatctggaaaagcgtccgc

23sapBC (showing *sapC* CDS) upper case: promoter (BPROM score 1.38)

2309805 TTGTCAacgtaatttccgaTATTCTcggtgccatggctaacccactgaaacat

fM P Y D S T Y K I I I F V N *

2309752 aaggaatggtATGCCCTACGATAGCACCTACAAAATAATTATTTTTGTCAATTAAaaa

int underline: Pint BPROM 2.78 upper case: PsapCisland BPROM 2.24

2286590 …CATcagtgtacacattttggttaaattgataaccTTCAAAtcaatgcagaacacaCAAAATtaaagcactcaatgctg-[32 bp]-

fM P Y D S V Y L E K R P P G A …

2286482 aagacttaaagcatgactaacaaacttaaaacggattcatATGCCCTACGATAGCGTTTATCTGGAAAAGCGTCCGCCCGGCGCGCT…

40guaA …G I S R V V Y D I S G K P P A T I E W E *

4010416 …GGTATCTCCCGCGTGGTGTATGACATCAGCGGCAAGCCGCCAGCAACGATTGAGTGGGAATGAttaacggctaccttatagctaatc

W E *

3969950 tcgtttcataaacttgttttcagtcaattggttacgagtgctgtaaataattgaGTGGGAGTAAtccccggcgttagctgaatgaaac

16fis …G I N R G T L R K K L K K Y G M N *

4919291 …GGCATCAACCGCGGTACGCTGCGTAAAAAACTGAAAAAATACGGCATGAACTGAtacatttcagctatgttattgaataaaaaggcg

N *

4935286 aaaacccactccaaagcatgaaaacagctaatctgacagtacggcaTGAACTGAtactagtcagttgcttgcttgatataaaaggcg

Fig. S3. Calibration curves for plasmid resistance to exonuclease. Plasmid read counts were normalized to total read counts, and values for Experiment X2 exonuclease-treated samples were divided by those for their untreated partner samples. Note the logarithmic y-axis. Slopes are similar, while intercepts differ, perhaps due to differing extents of DNA shearing during genomic DNA preparation. These curves predict enrichment factors for plasmid-like mobile DNA circles over a large size range.


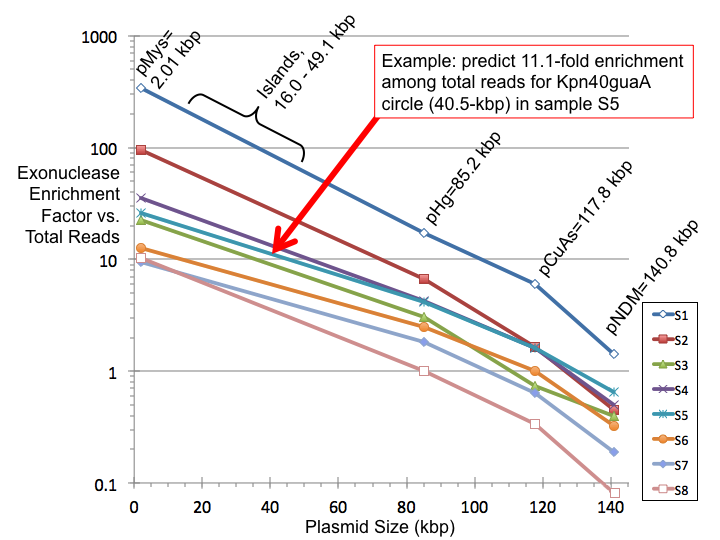


Fig. S4. IS CJs. CJ linker sequences detected by AttCt are given with their read counts for five IS types (the more abundant and varied IS*4321*and IS*5075* CJ sequences are summarized in Fig. 5). Upper case, IS sequence; lower case, flanking sequence; underlined, apparent base substitutions; brackets, additional sequence truncated for display. In most cases, the junction sequence could identify the source copy of the IS, which is presented above each CJ group with the total read count for that copy. Color coding indicates where source flank could be determined. Symbols: parenthesis, read counts; *, read was found by Juxtaposer, but a base change prevented AttCt detection; †, this case confirmed that both ends of the CJ came from the same IS copy, because the right end of copy IS*26*.6 bears a unique base substitution mutation G789C (not shown) and its left flank was captured in the CJ linker; ‡, the only IS CJ reads found in Experiment X0 (whose sequences were also unique to that experiment). [Figure continues on next page.]

IS*26*, 8 bp DR (27)

IS*26*.1 tgaaaaccgccactgGGCAC...GTGCCctggccgatgaagtt chromosome/2297523-2296704 (19)

ACTTTGCAACAGTGCCctggcc-----------------------------------------GGCACTGTTGCAAATA (2)

ACTTTGCAACAGTGCCctggccgat--------------------------------------GGCACTGTTGCAAATA (1)

ACTTTGCAACAGTGCCctggccgatgaagttaa------------------------------GGCACTGTTGCAAATA (1)

ACTTTGCAACAGTGCCctggccgatgaagttaataa---------------------------GGCACTGTTGCAAATA (6)

ACTTTGCAACAGTGCActggccgatgaagttaataa---------------------------GGCTCTGTTGCAAATA (1)

ACTTTGCAACAGTGCCctggccgatgaagttaatta---------------------------GGCACTGTTGCAAATA (1)

ACTTTGCAACAGTGCCctggccgatgaagttaataacggcttgtttgccagagttcttttt--GGCACTGTTGCAAATA (1)

ACTTTGCAACAGTGCC-----------------------------------------ccactgGGCACTGTTGCAAATA (3)

ACTTTGCAACAGTGCC----------------------------------------gccactgGGCACTGTTGCAAATA (1)

ACTTTGCAACAGTGCC--------------------------------------ccgccactgGGCACTGTTGCAAATA (1)

ACTTTGCAACAGTGCC------------aaaacagtcataacaagccatgaaaaccgccactgGGCACTGTTGCATATA (1)*

IS*26*.2 catagtcgagattggGGCAC...GTGCCggttttcgaggatat chromosome/2301833-2302652 (1)

ACTTTGCAACAGTGCCggttttcgaggatatttccagcgggaagaa-----------------GGCACTGTTGCAAATA (1)

IS*26*.3 tggcaggcttttcccGGCAC...GTGCCcgccgagctatggtg pCuAs/12327-13146 (4)

ACTTTGCAACAGTGCCcgccgag----------------------------------------GGCACTGTTGCAAATA (2)

ACTTTGCAACAGTGCCcgccgagctatggtgcagcgatcaccgatttgaagc[30bp]-----GGCACTGTTGCAAATA (1)

ACTTTGCA---------ccttagctcggcgctttgtcggtattgagcgtggcaggcttttcccGGCACTGTTGCAAAT (1)*

IS*26*.4 ccgtgctcgtgctgcGGCAC...GTGCCggagtgattcactgt pCuAs/13688-14507 (2)

ACTTTGCAACAGTGCCggagtgat---------------------------------------GGCACTGTTGCAAATA (1)

ACTTTGCAACAGTGCCggagtgattcactgtcaaagaatcggcccggtgctctgacgcaag--GGCACTGTTGCAAATA (1)

IS*26*.5 ctacgggctttttcaGGCAC...GTGCCggattgaatataacc pCuAs/18297-17478 (0)

IS*26*.6 gtagttaattttttgGGCAC...GTGCCcgctcagctggttgg pHg/14157-14976 (1)

ACTTTGCAACAGTGCC----------------------------------------ttttttgGGCACTGTTGCAAATA (1)†

IS*26*.7 ttgctgccgcctggaGGCAC...GTGCCgaaaattgcccgtac pHg/21817-20998 (0)

IS*26*.8 -----[partial]------...GTGCCcaaagaactggatat pHg/32883-33132 (0)

IS*26*.9 gaataacccggcgttGGCAC...GTGCCtttaagcgtgcataa pHg/37245-38064 (0)

IS*26*.10 ctatacctatcgagaGGCAC...GTGCCccacatcttttgtca pHg/41070-40251 (0)

IS*26*.11 atccccgaaaaatagGGCAC...GTGCCaatgtgggcgtctgg pHg/44490-43671 (0)

IS*26*.12 attgagaacaaaaacGGCAC...GTGCCaaaatatcgtgccag pHg/85160-84341 (0)

IS*Kpn14*, 9 bp DR (8)

IS*Kpn14*.1 ataccagacatttttGGTGA...TTACCcaagttgccatgtca pNDM-US/121200-121967 (2)

ATTGGAGTCATTACCcaagttgc-----------------------------------------GGTGATGCTACCAAC (1)

ATTGGAGTCATTACC-----------------------------------------acatttttGGTGATGCTACCAAC (1)

IS*Kpn14*.2 tcagtttggcgtgcgGGTGA...TTACCctcgccgcgttgttt pHg/81712-82479 (6)

ATTGGAGTCATTACCctcgccg------------------------------------------GGTGATGCTACCAAC (1)

ATTGGAGTCATTACCctcgccg------------------------------------------GGAGATGCTACCAAC (1)

ATTGGAGTCATTACCctcgca-------------------------------------------GGAGATGCTACCAAC (1)

ATTGGAGTCATTACCctcgcc-------------------------------------------GGTGATGCTACCAAC (1)

ATTGGAGTCATTACC-----------------------------------------ggcgtgcgGGTGATGCTACCAAC (1)

ATTGGAGTCATTACC-[50bp]agccataaacgacacgctggaaacgtatcagtttggcgtgcgGGTGATGCTACCAAC (1)

IS*Kpn18*, 3 bp DR (50)

IS*Kpn18*.1 aatcgcgaggtacagTGTTG...CAACAcagctcgttgagcaa chromosome/1445374-1446676 (5)

ACTTGACCACAACAcagTGTTGTGGTCAAAT (4)

ACTTGACCACAACAcagAGTTGTGGTCAAAT (1)

IS*Kpn18*.2 gcgcttaacgctgacTGTTG...CAACAgacaaacagccgctg chromosome/925510-924208 (45)

ACTTGACCACAACAgacTGTTGTGGTCAAAT (44)

ACTTGACCACAACAggcTGTTGTGGTCAAAT (1)

ISKpn1, 3-4 bp DR (31)

IS*Kpn1*.1 gcccgcgcaagcgtaTGGAC...GTCCAgtagcgccgccgggc chromosome/393395-391951 (9)

ATGGGGTCAGTCCA--gtaTGGACTGCACCCCA (6)

ATGGGGTCAGTCCA--gttTGGACTGCACCCCA (1)

ATGGGGTCAGTCCA-cgtaTGGACTGCACCCCA (2)

IS*Kpn1*.2 gcccggtaagcgcagTGGAC...GTCCAgcagcgccaccgggc chromosome/1456494-1457938 (3)

ATGGGGTCAGTCCA--cagTGGACTGCACCCCA (2)

ATGGGGTCAGTCCA-gcagTGGACTGCACCCCA (1)

IS*Kpn1*.3,5 gcccgcgcaagcgcaTGGAC...GTCCAgcagcgccgccgggc chromosome/2191706-2193150 (13)

chromosome/5179038-5177594 .

ATGGGGTCAGTCCA--gcaTGGACTGCACCCCA (12)

ATGGGGTCAGTCCA-cgcaTGGACTGCACCCCA (1)

IS*Kpn1*.4 gcccggctgcgctgcTGGAC...GTCCAtgcgcttgcgcgggc chromosome/3707641-3706197 (6)

ATGGGGTCAGTCCA--tgcTGGACTGCACCCCA (6)

IS*Kpn21*, 5 bp DR (447)

Unassignable (1)

ATTGAGCCTTGACA------TGTAATGGCTCAAT (1)

IS*Kpn21*.1 tatgcgtactcatgtTGTAA...TGACAcatgttgtagtagct chromosome/2729608-2731885 (145)

ATTGAGCCTTGACAc-----TGTAATGGCTCAAT (129)

ATTGAGCCTTGAAAc-----TGTAATGGCTCAAT (1)

ATTGAGCCTTGACTc-----TGTAATGGCTCAAT (4)

ATTGAGCCTTGACAca----TGTAATGGCTCAAT (5)

ATTGAGCCTTGACAct----TGTAATGGCTCAAT (1)

ATTGAGCCTTGACAcat---TGTAATGGCTCAAT (1)

ATTGAGCCTTGACAcatgt-TGTAATGGCTCAAT (4)‡

IS*Kpn21*.2 tatgcgcacctgtatTGTAA...TGACAaacattgtcagcaca pCuAs/48260-45983 (301)

ATTGAGCCTTGACAa-----TGTAATGGCTCAAT (278)

ATTGAGCCTTGACTa-----TGTAATGGCTCAAT (11)

ATTGAGCCTTGACAa-----AGTAATGGCTCAAT (2)

ATTGAGCCTTGACAa-----TCTAATGGCTCAAT (1)

ATTGAGCCTTGACAaa----TGTAATGGCTCAAT (4)

ATTGAGCCTTGACAaac---TGTAATGGCTCAAT (1)

ATTGAGCCTTGACA-----tTGTAATGGCTCAAT (2)

ATTGAGCCTTGACA-tgtatTGTAATGGCTCAAT (2)‡

Fig. S5. Matched transposition junction pairs. The interrupted site and gene context are given for the 14 cases where both ends of a single transposition event could be identified. Experiment and sample are given (‘d’ subscript denotes exonuclease treatment) with read counts for each left and right junction sequence. Upper case, target site sequence. Lower case, IS sequence. Blue, the direct repeat (DR) of the target site at the two ends of the IS. Red, the left end of the IS. Green, the right end of the IS. Note that both orientations (polarity inversion) of ISKpn14 were detected at the identical site in *cspC*, in the same sample. The underline shows the strongest case of IS/target match. Asterisk: The reads involving the chromosome:3043993 region are compatible with transposition into the chromosome of a 64240-bp segment of pHg, which is flanked by IS*Kpn14* at one end and the related element IS*1X4* at the other.

Chromosome:2376497, 8-bp DR, IS*26*Fwd in hypothetical (within Kpn42yraA)

Exp2/30 1 GACTACAAGAAAGCAGTTTACCGggcactgttgcaaat

Exp2/60 1 ctttgcaacagtgccGTTTACCGGATAGCAAGGCATGA

Chromosome:3043993, 9-bp DR, IS*1X4*/IS*Kpn14* Hybrid*Fwd in tRNA/hypothetical intergene

X2/S6,2 2 TTGTGTATTGGCAAAGCGTTTTCCggtaatggtgccaac

X2/S6,7 2 attggagtcattaccGCGTTTTCCTTGCATAGTCCATAC

Chromosome:3205506, 8-bp DR, IS*Kpn14*Fwd in *ppsA*

X2/S1 1 CATTGTTGGACATCGAGACAATCggtgatgctaccaac

X2/S3 1 attggagtcattaccAGACAATCCTTTTGTGATTTAAT

Chromosome:3398646, 9-bp DR, IS*Kpn14*Fwd AND IS*Kpn14*Rev with polarity inversion, in *cspC*

X0/S1 1 AAACCAAAACCTTTAGACTCGTTGggtgatgctaccaac

X0/S1 1 attggagtcattaccGACTCGTTGAACCACTTAACTTGA

X0/S1 1 AAACCAAAACCTTTAGACTCGTTGggtaatgactccaat

X0/S1 4 gttggtagcatcaccGACTCGTTGAACCACTTAACTTGA

Chromosome:3458139, 9-bp DR, IS*Kpn14*Fwd in *araF/ftnA* intergene

X2/S1 1 ATCTTTGGCGTAATTTATTGCGAAggtgatgctaccaac

X2/S3 1 attggagtcattaccTATTGCGAAAAGAAAAAATATATT

Chromosome:3570508, 9-bp DR, IS*Kpn14*Fwd in *wbaP*

X0/S1 8 TACGTGAAACTGGTACTAGTATGAggtgatgctaccaac

X0/S1 4 attggagtcattaccCTAGTATGATTATTAACAACCAAG

Chromosome:3570835, 9-bp DR, IS*Kpn14*Fwd in *wbaP*

X1/S1,2 13 AATCTGAAAAGGCAAGAGCGGCGCggtgatgctaccaac

X1/S1,2 12 attggagtcattaccGAGCGGCGCTGACGATAAGGTTTC

Chromosome:3971689, 8-bp DR, IS*26*Fwd in hypothetical (within Kpn40guaA)

X1/S1 1 TCAACCGCTGAGGTGACAAATATggcactgttgcaaat

X1/S1 1 ctttgcaacagtgccACAAATATTACCGCCCCAGCGAG

Chromosome:4744392, 9-bp DR, IS*Kpn14*Fwd in *dhaB*/hypothetical intergene

X2/S3,2 3 CAATAGCGGAACTTTAAATGAGCCggtgatgctaccaac

X2/S6 1 attggagtcattaccAAATGAGCCAGTGGTGAAAAAAAT

Chromosome:5311726, 9-bp DR, IS*Kpn14*Fwd in *yahK*/hypothetical intergene

X2/S2 1 TGAGAATCATAGTATCAACAGGTGggtgatgctaccaac

X2/S3d 1 attggagtcattaccCAACAGGTGAATTAACGAATAAAA

Plasmid pCuAs:23777, 8-bp DR, IS*26*Rev in hypothetical

X3/S9 1 TGTGATGACAATGCGCGATGAAGggcactgttgcaaag

X3/S9 1 atttgcaacagtgccCGATGAAGCGCCTGAAGCTTTCG

Plasmid pMYS:1889, 9-bp DR, IS*Kpn14*Fwd in its ORF1 gene

X2/S8d 1 AGCAAGTAAAAAAAACGGAATGCAggtgatgctaccaac

X2/S7d 1 attggagtcattaccCGGAATGCAAAGTAATAATGTTTT

Plasmid pMYS:1891, 9-bp DR, IS*Kpn14*Rev in its ORF1 gene

X2/S7d 1 CAAGTAAAAAAAACGGAATGCAAAggtaatgactccaat

X2/S6d 1 gttggtagcatcaccGAATGCAAAGTAATAATGTTTT

Fig S6. Transpositions into plasmids. Transposition sites pooled from exonuclease-treated and -untreated samples were jitter-plotted along each plasmid. Arrows mark the native copies of the main IS families (native copies of IS*Kpn18* are on the chromosome). The given **m**-value is for the lowest significant cluster size, corresponding to the bottom of the set of black horizontal lines, which map connected components of significant clusters with increasing **m** upwards.


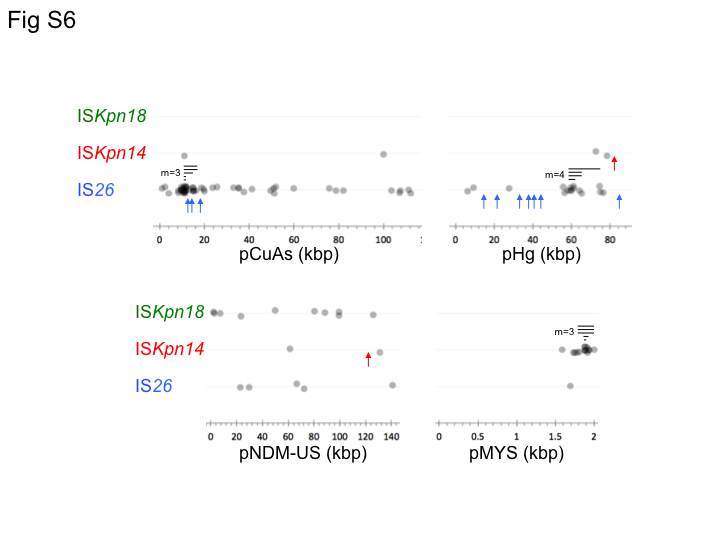


Fig. S7. IS*26* transpositions into repetitive regions. A) Mapping transpositions of IS*26* into IS*26*. In junctions marked above the line, the IS*26* end aims downstream; those below aim upstream. All were from exonuclease-treated samples except the case marked ‘Un’, suggesting they are circular foms. Ten of 13 are consistent with circle formation of an IS*26* fragment. One exception (*) was found to involve two IS*26* copies, with the uniquely marked right end of copy 6 invading partial copy 8 (red). For the other events mapped on the gray line, the target IS*26* copy could not be identified. White box: IS*26* left end; black box, IS*26* right end. B) Whole or partial identical copies of IS*6100* (color coded) lie near some IS*26* copies (black arrows). C) IS*26* transpositions into IS*6100*. Some could be resolved to IS*6100* copy, while those in the grayed central portion could not. Inv: polarity inversion of IS*26*.


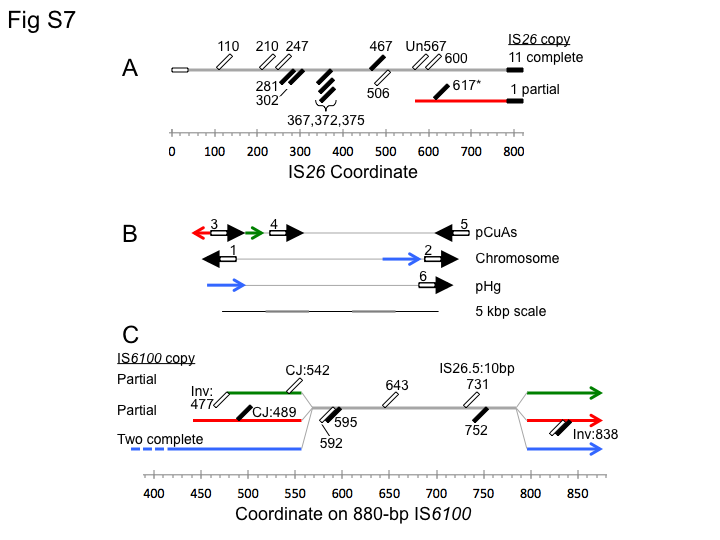


Fig. S8. Configuration breakdown of IS*26* transposition junctions mapped to the chromosome. Transposition junctions from samples untreated (panels A, C) or treated (panels B, D) with exonuclease, were sorted into the four configurations: left (L, red) or right (R, blue) IS*26* end, aiming upstream (+) or downstream (‑). Transposition junctions involving the uniquely marked right end of IS*26* copy 6 on pHg are shown in black and circled. Native IS*26* copies are numbered with left and right ends color coded at the top. Panels C and D enlarge the region of the native IS*26* copies, also marking genomic islands and a flanking antibiotic resistance gene in the vicinity. Panel D shows a summary of the configurations expected in different zones for *circles* arising from IS*26* copy 1 or copy 2 (see Fig. S9, C).


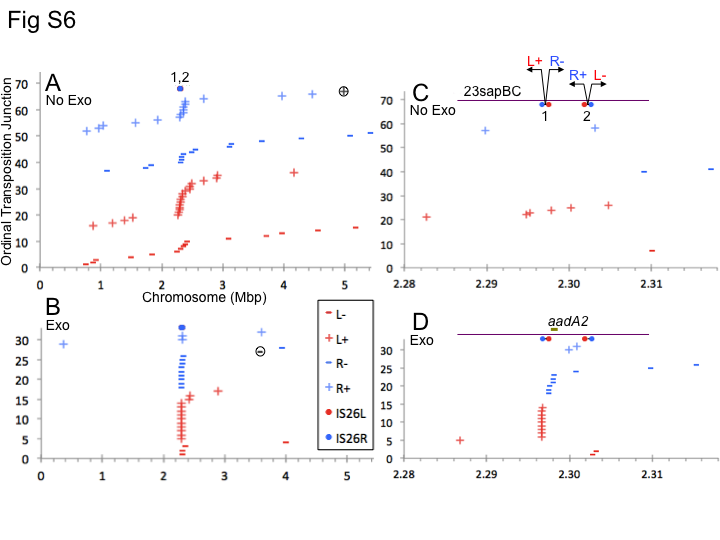


Fig. S9. Consequences of local transposition by IS*26*. A) Replicative transposition by IS*26* (blue arrow) into its same replicon leads (depending on the configuration of the attack) to either i) inversion of the IS together with one flanking sequence (green-to-red gradient), or ii) concomitant formation of both an IS circle with flanking sequence as a linker and a deletion of that linker sequence within the replicon ([13](#_ENREF_13)). Pink, the direct repeat of target sequence. Juxtaposer classifies reads from the IS circle linker as a CJ if the linker is short enough to be fully contained within the read or as a transposition if the linker is too long to detect both ends of the IS. B) Attack of an IS on a tandem neighbor (or homologous recombination between the two IS copies) can produce a circular molecule, but no new short range sequences are produced, such that Juxtaposer would reject all its reads as standard genomic sequence. C) The two chromosomal IS*26* copies (1 and 2) are separated by 4.3 kbp and in opposite orientation (nominal left ends in red, right ends in blue). If inversion and deletion/circularization events occur equally, yields of the four transposition junctions (L-, R+, L+, R-) should be balanced, but if circles are enriched (e.g., after exonuclease treatment), different neighborhoods of the two IS copies would have different yields of the four configurations.


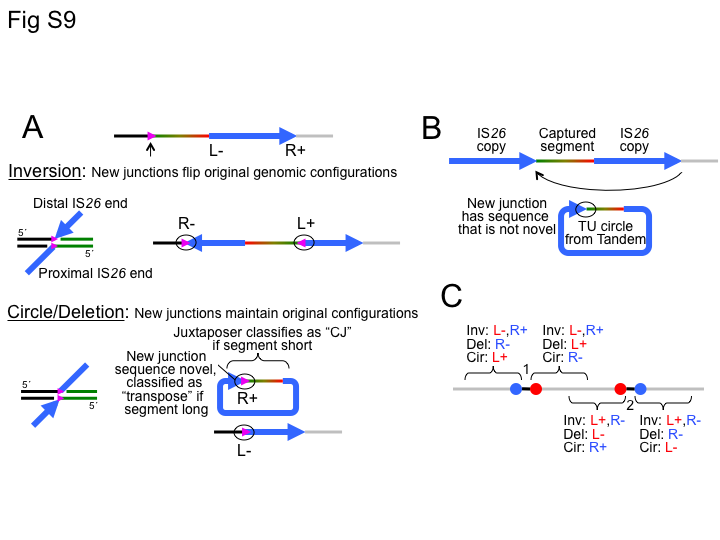


Fig. S10. IS*26* transposition junctions mapped to pCuAs. Conventions as in Fig. S8.


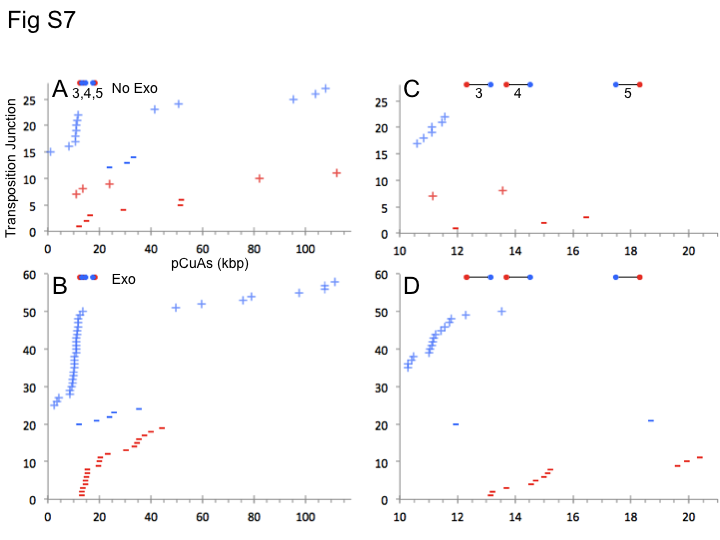


Fig. S11. IS*26* transposition junctions mapped to pHg. Conventions as in Fig. S8.


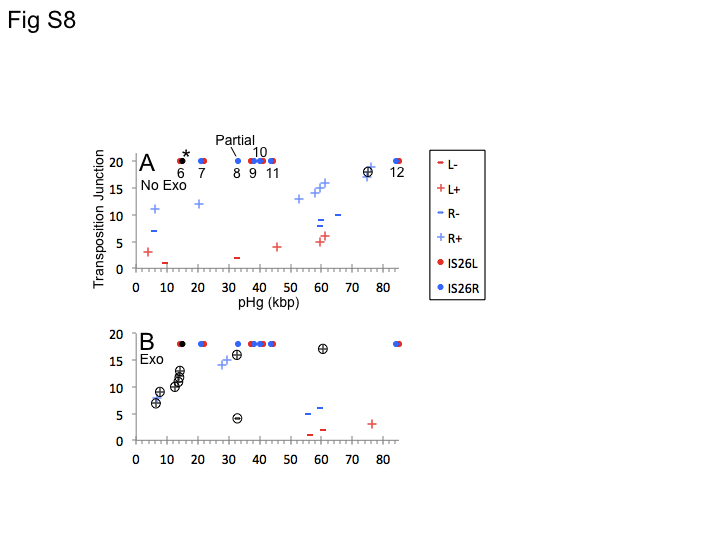

Supplement: SUPPLEMENTARY DATA [file supp_gkw601_nar-01118-h-2016-File008.docx]
